# Abstracts and Gleanings

**Published:** 1878-04-20

**Authors:** 


### *Abstracts and Gleanings.*

### IODOFORM.

M. Cuffer, in *La France Medicale*, speaks highly of the therapeutic effects of iodoform as an external application. He states that although no very appreciable benefit has followed its internal administration, its topical influence is very evident. Iodoform has a double action—anæsthetic and cicatrizing. Its anæsthetic properties render it useful in anal fissures, hemorrhoids, ulcerations of the throat and ulcerated cancers, especially those of the face, mouth, breast, and cervix uteri. It is necessary to use the remedy in fine powder, and to apply it carefully to all the diseased surface. The simplest way to obtain it in fine powder is to dissolve it in ether and allow the latter to evaporate. In using it for hemorrhoids it should be made into suppositories. It can be applied without danger in considerable doses, no bad effects having resulted from its use.

Its cicatrizing action is astonishing in its rapidity. Soft chancres, ulcerated buboes, mucous patches, and syphilitic ulcers of any kind, yield to it. Phagadenic ulcers are often arrested in their course, and onychiæ are cured in a few days. Scrofulous sores, lupus and epitheliomia of the lip have shown remarkable amelioration after its application. Inflammatory symptoms disappear, and exuberant granulations lose their unhealthy aspect, the progress made towards cure in a single day following the use of iodoform being often astonishing. Its penetrating odor is a great objection to its use, but nothing that has been tried as a substitute has given corresponding results. Its application requires certain precautions. The first is to apply it after thoroughly cleansing the wound. This may be done with the spray of warm water. Then the powder is applied and the wound covered with lint, the dressing being changed daily or twice a day at first, the intervals being gradually lengthened as the cicatrization progresses. It may be applied to the throat, or to the neck of the uterus, by dissolving it in ether and using the spray apparatus. (Tannin is said to disguise the smell of iodoform.)—*Canadian Journal of Medical Science.*

### TANNIN IN A CASE OF VERY INTRACTABLE VOMITING DURING PREGNANCY.

Diboue reports a case of this kind in the *Archives de Toxicologie* for September, 1878. The patient was a young woman, about 22 years of age, whose constitution was not very strong, but who had never had any serious sickness. The vomiting commenced very early in her pregnancy, but only became alarming after two months. All the usual means —such as iced drinks, alcoholic liquors, champagne, bitters of various kinds, antispasmodics, tonics, opiates, bromide of potassium, chloral, belladonna, etc.— were tried without benefit. Before resorting to the induction of abortion, it was determined to try tannin, which was given in the form of a pill, and in the dose of 1½ grains morning and evening. Two hours after taking the first pill, the patient was able to take a little soup, and this she did again in the evening. The vomiting was not entirely relieved, but was lessened to such a degree that the nourishment could be taken and retained in sufficient quantity. An intense headache, which had lasted for some weeks, also disappeared in a few days, and strength was rapidly regained.—*Wm. C. Dabney, M. D., Charlottesville, Va., in Virginia Medical Monthly.*

### THE IODIDE OF ETHYL IN THE TREATMENT OF ASTHMA.

Tuesday, January 29, Prof. German See communicated to the Paris Academy a full report of his treatment of asthma by the iodide of potassium during the intervals and the iodide of ethyl during the attack, from which we make the following extract regarding the latter agent. The report is published in full in the *France Medicale*, Feb. 2, 1878.

*Chemistry.*—The iodide of ethyl, discovered by Gay Lussac in 1825, is a mixture of two parts by volume of alcohol and one of hydriodic acid. This ether, devoid of any reaction whatever, has an odor like chloroform, a piquant taste and a density of 1.92 to 2.2 It is volatile, boils at 64° C., without being inflammable and under the influence of air browns a little, due to some iodine set free. Its formula is C.H.I in equivalents (Berthelol) its atonic formula being C. H. I. (Wurtz.)

*History.*—For twenty-five years it was forgotten until Huette with the design of using it as a substitute for iodine, which Laennec, Berton, Piorry, Scandamore and Murray administered by inhalation in the treatment of pulmonary phthisis, began to experiment with it upon himself and upon one of his friends.

The following are the effects observed after the inhalation of this ether mixed with air and collected with several millimetres of water in a flacon four centimetres in height.

After several inspirations the water is displaced and the ether is inspired. "It soon produced in the experimenter an impression of calm and comfort; the respiratory movements are executed with facility and amplitude at once. A surcharge of vigor is observed in all the muscles, appetite develops, the secretions become active, the pulse acquires plenitude, sensations become keener and the intellectual activity increases."

*Physiology.*—The following are the results which I have observed in cases of patients suffering with dysponœa whom I caused to inspire 6 to 10 drops of the iodide of ethyl 6 to 8 times a day:

In the healthy individual we observe at the end of several seconds a greater facility of respiration, and this phenomenon persists for several hours.

There is no anæsthetic or soporific effect whatever.

The heart and the circulation are not modified, although absorption takes place immediately almost, for iodine may be recovered from the urine in ten minutes.

Not infrequently there supervenes at the commencement of inhalation an access of cough.

*Therapy.*—I have employed this agent in five cases of asthma and the attack in all was rapidly arrested; in one case more rapidly than after nitrated fumigation or chloroform. In three cases of cardiac dyspnœa I have likewise remarked favorable results.

I have prescribed it also in three cases of chronic bronchitis accompanied with dyspnœa and the effect, though much less prompt, was nevertheless good.

Lastly, eight days ago I had occasion to prescribe it in a case of œdematous laryngitis in a patient aged 40, who was sent to me by M. Collin, our skillful fabricator of surgical instruments. For two days I hesitated over tracheotomy on account of threatened asphyxia and aphonia, but the patient recovered under inhalations repeated ten to twelve times a day.—*British Med. Journal.*

---

### MEDICAL APHORISMS.

The following dogmatic form of stating facts greatly simplifies the logic of conviction. They were picked out of Dr. Bartholow's *Materia Medica* by the *American Practitioner:*

"Quinia performs its offices by means of its antiseptic powers; is an antiferment. It may produce permanent deafness. It arrests inflammation in its forming stage, and is excellent in scarlatina, variola, and rubeola."

"Alkalies, in the treatment of rheumatism, are losing ground; and quinia, blisters and cold baths give better results."

"A solution of common soda, freely applied, will often remove bromidrosis from the feet and axillary glands."

"The sulphites, vaunted by Polli, are of no avail."

"Blue Lick water (of Kentucky) is useful in abdominal plethora and obesity. Hemorrhoids and engorgement of the pelvic viscera are relieved by it,and excellent results are obtained from its prolonged use in glandular affections, hepatic, splenetic, uterine and prostatic."

"Numerous cases of spina bifida have been cured by injection of tincture of iodine."

"Mercury increases the flow of bile, not by augmenting its secretion, but by producing reflex constriction of the gall-bladder, mechanically forcing out the bile."

"Spare women, by warm baths and inunctions of oil, may acquire flesh and roundness of form."

"Phosphorus is the best remedy for impotence. Oleum phosphoratum consists of twelve grains of phosphorus, and one ounce of olive oil. Dose, five to ten drops."

"Fowler's solution, in drop doses before meals, arrests the vomiting of pregnancy, and also the vomiting of chronic gastric catarrh from alcohol. Enteric diarrhœa is cured by arsenic."

"Permanganate of potash relieves the condition in which lumbar pain, frequent micturition, and urine with profuse brick-dust sediment and intestinal indigestion, are associated symptoms."

"Chloride of gold and sodium in $\frac{1}{20}$ grain doses, thrice daily, will relieve nervous dyspepsia; prevents decline of sexual power, cures sterility, and likewise weak and ineffectual erections and diurnal seminal emissions."

"Chloride of gold, in $\frac{1}{20}$ to $\frac{1}{30}$ grain doses, produces remarkable improvement in chronic Bright's disease."

"Seminal troubles that are relieved by gold are aggravated by bromide of potash, and *vice versa.*"

"Alum is the best cure for lead colic, and relieves the pain and nausea more certainly than any other remedy."

"Digitalis possesses great utility in scarlet fever. It lowers the temperature, and maintains the action of the kidneys."

"Belladonna has real curative power in erysipelas, and without doubt has power to arrest lacteal secretion."

"Opium is the most important agent we possess in the treatment of various inflammations."

"Aconite is of the highest value in the eruptive fevers, especially in scarlatina. It lowers the temperature, promotes diuresis and diaphoresis, and checks nasal, faucial and aural inflammations. In measels it arrests the catarrhal pneumonia. In idiopathic erysipelas we have no better remedy; and in cerebral and spinal meningitis, prior to effusion, aconite is as serviceable as in other inflammations."

"Salicylic acid in typhoid, erysipelas, acute rheumatism, pneumonia, phthisis, etc., is only second to quinia as an antipyretic. For intermittents it seems nearly, if not quite equal to quinia."

"Acu-puncture is so decided in its relief of pain, that some physicians contend that the anodyne effects of hypodermic injections of morphia are due to the water, and not to the opiate. The injection of water, to be efficacious, must be near to the seat of pain. In facial neuralgia, sciatica, lumbo-abdominal neuralgia, lumbago, uterine colic, and irritability of the bladder, acua puncture possesses extraordinary power. In paralyzed and wasting muscles, it promotes nutrition of the muscles and contributes to the regeneration of muscular power. Thirty to sixty minims of water should be injected at the painful points; and if no relief occurs in two minutes, repeat the remedy."

"An ingenious use of bicarbonate of sodium to produce emesis, applicable in narcotic poisoning. Sufficient soda is first swallowed, and immediately after a suitable proportion of tartaric acid is taken. Brisk effervescence ensues, thoroughly emptying the stomach."

"Arsenic may cure epithelioma; is useful in scirrhus, and palliative in uterine cases."

"Eucalyptus, though an unequaled remedy in catarrh of the bladder, is a very inferior antiperiodic."

"Hydrastin stands next to quinia as an antiperiodic; is useful in all the conditions in which quinia is used, and is an excellent injection for gonorrhœa."

---

### POLYPUS OF THE UTERUS.

A polypus is a pedunculated tumor of the womb. In addition to the polypus there are three kinds of fibroid tumors—the mural, submucous, and subperitoneal myoma of the womb. Some physicians divide polypi of the womb into four varieties, but there is practically no difference between them. A polypus is very unlikely to return after it has been removed. Polypi are sometimes multiple. The more open is the os uteri, the easier it is to remove a polypus. In making your diagnosis of a polypus be sure not to confound it with an inverted womb. The ecraseur has very often been applied to an inverted womb, mistaking it for a polypus. A polypus is insensitive, the womb is highly sensitive. When the tumor is an inverted womb, a rectal examination will always show a cupped depression. Always pass your sound before attempting to cut off a polyp. Supra-pubic palpation will reveal the womb present in, or absent from, its normal position, as the case may be. It is sometimes extremely difficult to get at the pedicle of a polyp. Here I cannot get the polypus altogether out of the uterine cavity, but I can distinctly feel its pedicle. The womb is not quite two and a half inches long. I think the polyp has pulled the fundus down slightly.

I intend to use the wire ecraseur in this case. The wire may break, but I am prepared for that accident. I fasten both ends of the wire to the travelling button, so as to get a crushing action. I am using for my loop a piece of piano wire; wire used for producing the upper notes of the piano. The tumor is a large one, and, as you see, the bleeding from its delicate surface is extensive. If a polypus fills up the vagina completely, so much so that you cannot find room to apply the ecraseur, you may either (1) deliver it with the forceps, as you would the head of a child, or (2) you may put the ecraseur around as large a portion of it as possible, and so remove it piecemeal. Some years ago I had to perform three operations to get the whole mass away. Be very careful that you do not catch some part of the womb with your encircling wire. You see how easily I have brought this growth away. I have never had a single bad symptom follow this operation in my hands; of course, this has been a piece of good luck.—*Medical and Surgical Reporter.*

---

### CARBOLATE OF SODA IN THE TREATMENT OF NERVOUS AFFECTIONS OF THE RESPIRATORY PASSAGES.

According to Dr. Pernot, all nervous and spasmodic affections of the bronchi—asthma, catarrh, influenza, and simple colds at the outset—are markedly influenced by the vapor of this raw carbolate of soda. On whooping-cough its action is most striking. He has found from numerous observations that after from two to ten days of treatment the paroxysms of coughing become much less frequent, less prolonged, and less severe, the vomiting diminishes or ceases, and respiration becomes easier. He has never seen the symptoms become worse after his treatment was begun. The treatment consists in volatilizing the liquid by means of heat in the sick-room two or three times a day. About ℥ x. of the liquid are placed in a porcelain vessel and exposed to the flame of an alcohol lamp. It volatilizes almost completely. The patient is allowed to breathe a purer atmosphere for two or three hours between each sitting, but a saucer containing some of the liquid is constantly kept under the bed. Dr. Pernot reports a few cases of pertussis and one of asthma, which illustrate well the action of the remedy, and certainly seem to justify all that he claims for it. Where a porcelain cup and an alcohol lamp cannot be got, a heated fire-brick may be used to volatilize the drug.—*Lyon Medical, Sept.* 23, 1877.

### VACCINATION WITH HORSE-LYMPH.

Prof. Demme reports several cases of vaccination in which humanized horse-lymph was used. The experiments were performed in the children's hospital at Berne, in Switzerland. Vaccination was always successful, with the exception of one or two punctures. Both the eruption and maturation of the vaccine pustules were delayed; the former from 1½ to 2 days and the latter from 3 to 5 days. All the pustules were very beautiful, and inflammatory reaction was much less than with the ordinary method of vaccination.

### TREATMENT OF CRACKED NIPPLES.

The success obtained by M. Cheron in the treatment of fissures of the anus with picric acid, induced M. Charrier to use the same application in the treatment of fissures of the nipple. He found that it relieved the pain and checked the morbid secretions in a very short time, while at the same time the delicate epithelium of the nipple became, as it were, tanned, and much less susceptible to alteration. In seven patients the application brought about a complete cure in from six to twelve days. The pain was relieved in from twelve to twenty-four hours, and nursing could be resumed without inconvenience to either mother or child. It is necessary that the picric acid used should be chemically pure, and completely deprived of soda. Two solutions are used, one containing 13 parts of acid to 1,000 of distilled water, and the other 1 part to the 1,000. The nipple should first be thoroughly cleansed with warm water and a fine sponge, and the concentrated solution then applied with a brush several times in succession to the fissures and all inflamed points. This is to be repeated every morning only, but after every nursing the nipple is to be held for three or four minutes in a small glass filled with the weaker solution.—*Gazette Meaicale de Paris, August 4th.*

### GLYCERINE IN THE TREATMENT OF INTERNAL HEMORRHOIDS.

Dr. David Young reports, in the *Practitioner* for January, a number of cases of internal hemorrhoids which he had successfully treated by means of glycerine, given internally, in doses of from one to three drachms, night and morning. He discovered this application of the remedy accidentally, from giving glycerine as a substitute for sugar to a patient who was suffering from internal piles. Subsequent trials of the remedy have convinced him that glycerine should be added to our list of palliatives for this troublesome malady.—*Mich. Med. News.*

NITRO-MURIATIC ACID should always be prescribed by itself. A damaging explosion occurred lately in a mixture ordered by a physician, containing that acid and tincture of cardamom.—*Phila. Med. Times.*

### TOXIC PROPERTIES OF DYNAMITE.

In a Paris thesis, M. Bruet sums up with the following conclusions as to the toxic properties of dynamite in nitroglycerine *Annadi* (*Universali di Medicina,* August.) 1. Nitro-glycerine is a poison, the energy of which is in direct proportion to the rapidity of its absorption. 2. It is most violent when quickly absorbed; a few drops are sufficient to strike down an animal in five minutes, and death follows in clonic and tonic convulsions. 3. It is less dangerous when absorbed slowly, and in this case kills by asphyxia, the fatal dose being rather high. A man exposed chiefly to the absorption of nitro-glycerine has rather to fear the chronic or slight results than acute poisoning or death. But he should avoid all conditions which may expose him to rapid absorption of the poison, as in this case there would be danger of sudden death. 4. For these reasons it is not superfluous to take precautions against exposure to an atmosphere in which particles of dynamite are given off.—*London Med. Record*, Nov. 15, 1877.

---

### MENSTRUATION AND OVULATION.

Dr. T. Gaillard Thomas, at a meeting of the New York Obstetrical Society (*American Journal of Obstetrics*, Oct. 1877), said that he had repeatedly diagnosed double ovarian tumor from the absence of menstruation, and the operation had shown the correctness of his opinion. He felt that the future would show that menstruation does depend on the function of the ovary.

Dr. Noeggerath said that one case of menstruation persisting after the ovaries had been removed would prove the lack of dependence of menstruation on the ovaries, and many such cases had been collected.

Dr. Thomas mentioned that he had removed both ovaries in ten cases. Two died; of the remaining eight, only one had menstruated since the operation.—*American Journal Medical Science.*

---

### TREATMENT OF EPILEPSY.

Dr. Schultz records in the *Berliner Klinische Wochenschrift* the case of a young man, eighteen years of age, the subject of epileptic attacks, which always came on at a certain hour in the day. It mattered not what he might at that time be doing, the attack never failed. It was always preceded by an aura which lasted five or six minutes, and was followed by a sleep of several hours' duration. Quinine in large and small doses, promide of potassium, strychnine, belladonna, nitrate of silver, morphia, chloral, etc., were all administered without result; the attacks continued to recur at the fixed hour, and even occurred during sleep induced by chloral. Coming at this time under Schultz's care, he determined to test Nothnagel's treatment, and administered a teaspoonful of ordinary salt during the aura. This did not at first prevent the attack, but when on the following day a heaping tablespoonful of salt was given at the very beginning of the aura, no attack took place. For one week the dose was administered at the usual time, although no aura was perceived. At the date of Schultz's report (seven weeks afterward) no attacks had been observed, though previous to the treatment, the patient had had them for 134 days in succession.—*St. Petersburger Med. Wochenschrift*, No. 4, 1878.

---

### THE DANGERS OF INFLAMMABLE VOLATILE LIQUIDS.

At Lyons, France, a young lady was about to undergo an operation requiring actual cautery. Anæsthesia was produced by ether, the vapor of which became ignited by the hot iron. The patient was badly burned about the face and mutilated for life. The *Lancet* argues that where thermo-cautery is used, chloroform should be employed rather than ether. Care should be taken even in the application of collodion near a naked flame. . . The galvano-cautery is frequently used while etherization is going on—whether safely or not remains to be demonstrated.

---

Excision of Larynx, and the use of artificial vocal apparatus, by Dr. Foulis, of Glasgow, is reported in the *British Medical Journal*, Dec. 8th. The operation was necessitated by malignant growth and gradual occlusion of the upper larynx. It was a success, and the patient had been enabled, by a hard rubber laryngeal tube, with vibrating reeds acting for vocal chords, to speak in a resonant, loud and clear, though monotonous voice. The *Lancet*, Feb. 2d, gives a cut, with description by Dr. Foulis, of the artificial larynx. . . This operation is said to be the first of the kind performed in Great Britain.—*Society County Kings.*

MALT EXTRACT AS AN EMULSIFIER.

Professor Geo. F. H. Markoe recently called attention to malt extract as an emulsive agent for cod-liver oils and other oleaginous preparations. At the present time, when cod-liver oil is extensively employed as a therapeutical agent, anything that will neutralize or overcome its disagreeable oily character and bad taste will be welcomed by patients. Extract of malt possesses the power of producing a perfect emulsion with cod-liver oil and extract of malt was exhibited, having a semi-solid consistence, in which the taste of cod-liver oil was more perfectly concealed than can be accomplished by any other known process.—*Boston Medical and Surgical Journal.*

BELLADONNA IN DYSENTERY.

Dr. Smith, of Cloverdale, Cal., recommends the use of belladonna in the treatment of dysentery, and states that he has obtained more satisfactory results with it than with any other drug. He frequently gives from two to four drops of the fluid extract every one, two, or three hours, until the griping pains are relieved. The full remedial effect is in some cases not manifest, until slight delirium or disturbance of vision is produced, but these symptoms disappear when the belladonna has been withheld for a few hours.—*Nashville Journal of Medicine and Surgery, August,* 1877.

OVULATION WITHOUT MENSTRUATION.

The relation of the discharge of ova to menstruation and of menstruation to the discharge of ova is a question to which considerable attention has in recent years been directed. It has been shown repeatedly by anatomical examination that menstruation may take place without the occurrence of ovulation, but similar evidence has hitherto been wanting in favor of the belief that ovulation may take place without menstruation. The opinion that ovulation may take place without menstruation has been based upon the fact that women who have never menstruated have borne children; but this was unsatisfactory, inasmuch as the objection may have been raised that the woman would have menstruated had not conception taken place; that, in fact, the occurrence of conception prevented that of menstruation. M. de Sinety has,however,set the question at rest by anatomical evidence. Before the Societe de Biologic he described the anatomical characters of the uterus and ovaries of a woman who had never menstruated. She was thirty-eight years of age, and, with the exception of the menstrual flow, had presented from her tenth year all the symptoms of puberty. The uterus was externally of normal volume, but the cavity was formed almost entirely by that of the neck; the cavity of the body was like that of the fœtal organ, and the mucous membrane presented the character of the infantile condition. Ovulation had been very active, for the ovaries presented many false corpora lutea.—*London Lancet.*

NITRITE OF AMYL AS AN ANTIPERIODIC.

Dr. W. E. Saunders writes to the *Lancet:*

Finding, as I did, that quinine did not give the satisfactory results I had been led to suppose from the current literature of the day, I was induced to try to find some drug that would act more surely and with greater effect than did quinine. This was more necessary since quinine was daily becoming scarcer, while the demand for it had increased. I tried nearly every form of treatment that I had heard of, but found nothing to rely on in most cases. After carefully comparing the cold stage of ague with the collapse stage of cholera and other diseases in part resembling it, I came to the conclusion that the collapse stage was practically the same in all, and that one form of treatment would accomplish what was required. I then found that nitrite of amyl was the remedy I wanted, and accordingly used it in two or three minim doses, by inhalation. The result was that I found I could remove the cold stage of ague in five or ten minutes, and that the hot and sweating stage was reduced in like proportion.—*Medical and Surgical Reporter.*

### GRINDELIA ROBUSTA IN WHOOPING-COUGH.

At a recent meeting of the Suffolk District Medical Society, Dr. Pattee called attention to the beneficial effects of the drug in certain pulmonary affections, and remarked that most of the fluid extracts sold in this market were said to be worthless. Dr. Pattee had used the tincture in bronchitis, asthma and whooping-cough, in doses half a drachm or more, repeated every one or two hours. The effect was said to have been curative in thirty cases of whooping-cough, after three or four days, without the occurrence of relapses. The dose for a child two years old would be about ten drops.—*Pharmacist.*

### A PRIZE OF $80,000.

The *Scientific American* calls the attention of our physicians and surgeons to the fact that there is a reward amounting to $80,000, left by will, for the French Academy of Sciences to give to the discoverer of a cure for cholera. The following are the particulars: The competitor is required: "(1) To point out a system of medicine that cures cholera in the immense majority of cases; or (2). To indicate, in an incontestible manner, the causes of Asiatic cholera, so that, by suppressing these causes, the epidemic will cease; or (3). To discover some certain prophylactic as evident for cholera; for instance, as vaccine is for the small-pox. (4). To become entitled to the annual prize (derived from *the interest* of the $80,000) the competitor will have to demonstrate, by vigorous processes, the existence in the atmosphere of substances that may play a part in the production or propagation of epidemic diseases; and (5). In case none of the above conditions have been fulfilled, a competitor may take the annual prize by finding a radical cure for tetter, or enlightening the world upon the etiology of that disease. Portions of the revenue have from time to time been awarded for meritorious essays."—*Jour. Mat. Med.*

### SUMMER COMPLAINT.

Dr. Mm. M. Gross, in brief, says: The very best remedy, in my judgment, for cholera infantum or summer complaint in children is calcined radix rhei.

My attention was called to it incidentally, during last August. I was treating a little patient, aged six months, affected with this dreaded trouble—had used all the reputed remedies for this disease, but with little or no effect. When my attention was called to it, I prepared some by putting a portion of the root in an iron vessel and burning it until it was easily pulverized. Of this I gave about five grains; the child became quiet and seemed free from pain, and in about three hours the bowels moved again, passing a changed and even larger evacuation than at any previous time; and from that moment it began to get better, and in a few days was entirely free from the disease. The success attained in this case led to the use of the same drug in a number of similar cases and with the same results.

In the forms of summer complaint incident to debility of the bowels, either when this condition depends upon general causes alone, or is the immediate effect of irritating ingesta or biliary derangement, rhubarb, in this form, is superior to almost every other medicine.

### BORAX AND NITRATE OF POTASSIUM IN SUDDEN HOARSENESS.

These two salts have been employed with advantage in cases of hoarseness and aphonia occurring suddenly from the action of cold. The remedy is recommended to singers and orators whose voices suddenly become lost, but which by these means can be recovered almost instantly. A little piece of borax the size of a pea is to be slowlo dissolved in the mouth ten minutes before singing or speaking; the remedy provokes an abundant secretion of saliva, which moistens the mouth and throat. This local action of the borax should be aided by an equal dose of nitrate of potassium, taken in warm solution before going to bed.—*La France Medicale.*

### STIBIODERMIC TREATMENT IN ACUTE ARTICULAR RHEUMATISM.

M. I. Guerin read a paper at a meeting of the Paris Academy of Medicine, upon the 4th of September, upon the above treatment of acute articular rheumatism by means of what he terms the stibiodermic method. This consists in the inunction every six or eight hours of an ointment containing one part of tartar emetic in two of lard. He states that a rapid reduction may thus be obtained of the pain experienced in the early stage of coxalgia. The cessation of the pain he attributes to a dynamic action of the remedy, and not to any revulsive action. The same results were obtained in gout when the symptoms only preluded the definitive attack, three or four inunctions being sufficient to prevent the onset of the disease. When, however, the attack was fully developed, a fly blister placed on the centre of the swelling quickly effected its removal. —*Archives generale de Medicine.*

### A HITHERTO UNRECOGNIZED SYMPTOM OF ISCHIATIC DISLOCATION OF THE HEAD OF THE FEMUR.

Some years ago, I observed that when the position of the dislocated limb to the body is changed, a marked difference occurs in its relative length. If the patient be placed upon his back and the thigh be flexed upon the trunk at a right angle, then the knee of the dislocated limb will sink below that of the other side from one to two inches. A moment's reflection will make this clear. The ischiatic notch is situated directly behind the acetabulum, the head being thrown from one to the other, the limb is shortened the distance from the centre of the cavity to the centre of the notch. This, in all cases, will be as much as one, it may be two or more inches.—*The Clinic.*

### PURULENT OPHTHALMIA OF INFANTS.

Dr. Luton, of Rheims, states that the tincture of iodine in distilled cherry-laurel water is a far more efficacious and innocuous means of treatment than the nitrate of silver. One gramme of the tincture may be added to twenty grammes of the water of medium strength (20°), and produces a collyrium the color of pale brandy. Some of this should be dropped into the eye four or five times a day, external lotions being also abundantly employed. It has proved rapidly successful at the Hotel-Dieu of Rheims.—*Revue Med.*

### FEEDING PER RECTUM.

An article appears in the *Deutsche Zeitschrift fur Praktische Medicin* (No. 44, 1877), in which Dr. Kauffmann draws attention to the excellent results he has obtained from the plan of feeding the patients with pancreas and meat in cases of persistent and incurable intestinal obstruction. He states that he has had nine patients in the Kolner Burger Hospital, several of whom were suffering from cancer of the œsophagus, one from cancer of the pylorus, and one from chronic ulcer of the stomach. In all of these a cleansing enema was administered in the morning, followed by the introduction into the rectum of a mixture of a pound of finely divided beef, and one-third of a pound of finely minced pancreas, the whole being freed from fat and connective tissue. Half of this quantity was used at noon, and half at 6 P.M. The results were excellent; a solid, well-formed evacuation was discharged every day. The patients were able to walk about, and lived for nine or more months.—*Lancet.*

---

STRYCHNIA IN BRONCHITIS.

In a letter to the *Philadelphia Medical Times*, of January 19th, Dr. Fothergill dwells at some length on the great value of strychnia as an expectorant in bronchitis. By its action on the respiratory centre, it proves useful when increase of respiratory power is needed for the expulsion of mucus gathered in the air-tubes. He gives it either alone or in combination with the ordinary cough mixtures. On the same principle it has proved useful in chronic bronchitis, with emphysema, and in the dyspnœa of advanced Bright's disease.—*N. Y. Med. Jour.*

BELLADONNA IN COLLAPSE.

Dr. Reinard Weber, in the *Philadelphia Medical Times*, recommends the use of small doses of belladonna as more efficient in cases of collapse than camphor, musk, alcohol, and other stimulants usually prescribed to restore the failing action of the heart. Dr. Weber claims to have been the first to recommend the use of belladonna for this purpose. He gives a physiological theory of its action, and supports his arguments by reports of several cases.—*N. Y. Med. Jour.*

THE TREATMENT OF BURNS BY BICARBONATE OF SODA.

One of the strongest pieces of testimony in favor of the use of bicarbonate of soda in the treatment of severe burns is that given by Dr. Burns, the editor of the *Philadelphia Medical Times.* One of the assistants burnt the inside of the distal phalanx of the thumb whilst engaged in burning some glass tubing. A saturated solution of the bicorbonate was at once applied; in five minutes the pain was gone, and with it all soreness, so that the part, although blistered, was freely used and pressed on in bending tubing, screwing up and unscrewing apparatus, etc. The relief was quick and complete, and the fact of its being witnessed by Dr. Wood himself makes it important that a remedy so simple and easily obtained as washing soda should be extensively tried.—*Lancet.*

THE REMOVAL OF MOLES.

These disfiguring growths, says a writer, may be best removed by the acid nitrate of mercury. The acid should be applied with a splinter of wood and gently rubbed into the part for several seconds, according to the thickness of the growth. Great care should be taken to prevent the acid reaching the surrounding skin. There is absolutely no pain attending the application, and the growth gradually shrivels away, and the slough falls off in about a week.—*Med. Rep.*

M. GUERIN'S TREATMENT OF CARBUNCLE.

The method consists in plunging the knife into the centre of the anthrax, and cutting away, under the skin, on to the limits of the diseased tissues, and even into the healthy tissues beyond. This is repeated four times, making a cross. The advantages of this method, according to M. Guerin, are considerable. They constitute the best method of treating anthrax, and far outstrip the two other methods, of open incisions or not cutting at all.

ERGOT IN DIABETES INSIPIDUS.

Prof. Da Costa, in a recent clinical lecture on the diabetes insipidus (*Hospital Gazette*), prescribed ergot, in half drachm doses of the fluid extract and three times a day. He was led to do this from the favorable result following the use of this drug in two previous cases. In these cases there was at once apparent decrease in the quantity of urine passed daily. The remedy was increased to two drachm doses, and the urine fell from ten pints to six pints, and finally to three.—*Mich. Med. News.*

